# Caffeine Use in the Anesthetic Management of a Patient With Congenital Central Hypoventilation

**DOI:** 10.7759/cureus.26646

**Published:** 2022-07-07

**Authors:** Jevaughn S Davis, Luca Allais, Claude Abdallah

**Affiliations:** 1 Department of Anesthesiology, George Washington University School of Medicine and Health Sciences, Washington, USA; 2 Department of Anesthesiology, Children’s National Medical Center, Washington, USA

**Keywords:** hypoxia and hypercapnia, abnormal ventilation, dysregulation of respiration, congenital central hypoventilation syndrome, caffeine use and anesthesia, caffeine use for apnea, caffeine usage

## Abstract

Congenital central hypoventilation syndrome (CCHS) is a rare neurological disease affecting the brain’s response to carbon dioxide levels, resulting in dysregulation of respiration. CCHS is characterized by a diminished effort to breathe during sleep despite hypoxia and hypercapnia. Ventilation is adequate during wakeful periods but diminished during sleep. Alterations in ventilation pose a challenge to anesthesiologists in their attempts to wean these patients from ventilatory support. We describe a patient with CCHS and a complicated history of prolonged tracheal intubation, who was treated with intravenous (IV) caffeine and was able to resume adequate spontaneous ventilation and baseline mental status immediately post-procedure.

## Introduction

Congenital central hypoventilation syndrome (CCHS), often referred to as Undine’s curse, is caused by sporadic, polyalanine repeat expansion mutations in the PHOX2B gene [1]. Its incidence is 1 in 50,000-200,000 live births. CCHS is inherited in an autosomal dominant pattern without a predilection for race or sex [1-2]. It is often associated with disease states that result from neural crest malfunctioning [1-5]. CCHS prevents the bodily detection of elevated carbon dioxide (CO_2_) levels, leading to failure to initiate spontaneous respiration, persistent hypoventilation, and hypoxia. This can result in delayed emergence, prolonged tracheal intubation periods, and unplanned intensive care unit (ICU) admissions or unplanned hospital stays. In the general pediatric population, delayed emergence and/or postoperative hypoventilation may result from residual muscular blockade, opioid-induced hypoventilation, drug-induced central nervous system depression, surgical/incisional pain, and increased CO_2_ production [3]. Pediatric patients diagnosed with CCHS are hypersensitive to narcotics, sedatives, and residual neuromuscular blockade, further exacerbating their risk of delayed emergence and/or postoperative hypoventilation [4].

CCHS usually presents in the newborn period with duskiness, cyanosis, and shallow breathing during sleep. Polysomnography will show normal breathing patterns while awake and a normal respiratory rate but shallow breathing with diminished tidal volumes while asleep. Desaturations and central apnea periods without observed obstructive episodes during non-rapid eye movement (REM) sleep are key features [1]. The hypopnea-apnea index will be elevated and can help characterize disease severity. A PHOX2B mutation is required to make the diagnosis [1]. Treatment may include tracheotomy, diaphragmatic pacing, and noninvasive positive pressure ventilation during periods of sleep [1-5].

We report the case of a patient with known CCHS undergoing general anesthesia. Previous anesthetic exposures were complicated by multiple prolonged ICU admissions due to the inability to wean from mechanical ventilation postoperatively due to severe hypoventilation. In this occurrence, tracheal extubation and resumption of adequate spontaneous ventilation were successful after the administration of IV caffeine toward the end of the procedure. The patient’s parents provided written consent and written HIPAA authorization was obtained for the publication of this case report.

## Case presentation

A two-year-old, 15.2 kg, ASA classification 3 male presented for combined procedures including dental restoration, dental extractions, exam under anesthesia, botox injection with colorectal surgery, and chest mediport removal with interventional radiology. Past medical history included congenital central hypoventilation syndrome, asthma, sleep apnea, seizures, Hirschsprung’s disease, and multifocal paraspinal neuroblastoma. The birth history was notable for full-term birth and hypoventilation requiring intubation for a period of one month and an overall pediatric intensive care unit (PICU) stay of over four months. He was not on any supplemental oxygen therapy at home and had no drug-related allergies, and his family history was negative for consanguinity and noncontributory to anesthesia complications. The patient's mom received standard antenatal care to her knowledge. Prior anesthetic records showed severe hypercarbia, wheezing, and hypoventilation. There was a history of inability to resume adequate spontaneous ventilation, even with the use of short-acting anesthetics and avoidance of muscle relaxation. Prolonged tracheal intubation periods and PICU stays were required following general anesthesia. The physical exam was unremarkable except for poor dentition. The patient was breathing at a normal rate in room air without distress. SPO_2_ was 98-100% and he had no dysmorphic features.

The patient was agreeable upon arrival in the operating room. He was connected to standard American Society of Anesthesiologists monitors with normal vitals. Anesthesia induction was achieved with the inhalation of 8% sevoflurane in oxygen. After the loss of consciousness and intravenous access was obtained, IV ketamine (2 mg/kg) and IV propofol (3 mg/kg) were administered to facilitate endotracheal intubation. End-tidal carbon dioxide (EtCO_2_) was noted to be 72 mmHg immediately postintubation. Anesthesia was maintained with a 45% oxygen/air mixture and end-tidal sevoflurane at 3%. The surgical time was 207 minutes, and vital signs remained stable throughout the procedures. Intraoperative medications included intravenous decadron (0.4 mg/kg), ondansetron (0.15 mg/kg), acetaminophen (15 mg/kg), and ketorolac (0.5 mg/kg). Premedication with midazolam, opioids, and muscular relaxation was purposefully avoided. Ventilatory settings were set to maintain EtCO_2_ in a range of 60-70 mmHg to avoid normalization. Surgeons were asked to inject local anesthetics in the field to avoid postoperative surgical pain.

After inhalation anesthesia washout, the patient was noted to be spontaneously breathing at a respiratory rate of 12 to 16 breaths per minute. However, tidal volumes were inadequate, and wheezing was noted in all lung fields. EtCO_2_ increased and peaked at 104 mmHg. Albuterol was administered for wheezing and the patient was placed on pressure support ventilation to avoid respiratory fatigue. A total of 120 mg of caffeine citrate was then administered intravenously in two consecutive boluses. EtCO_2_ improved to 62 mmHg. Within five minutes, the patient was able to maintain adequate tidal volumes while spontaneously breathing. His vital signs remained stable throughout the process, and his lung fields cleared. He was extubated to a nasal cannula and transported to the PICU as a planned admission given his prior post-procedural history. Within three hours, he was transitioned to room air and was discharged directly from the PICU to home the next day. This was his shortest PICU and hospital stay following general anesthesia to date.

## Discussion

CCHS poses a pharmacologic challenge to anesthesiologists given that the majority of commonly used anesthetics cause respiratory depression, potentially aggravating CCHS. Opioid administration induces respiratory depression, leading to hypercarbia and respiratory dysregulation. In fact, the literature points to CCHS patients being more sensitive to the depressant effects of opioids. Opioid sparing regimens, such as in this case, are recommended to improve outcomes [1]. Anesthetic technique usually revolves around the avoidance of preoperative sedatives, anxiolytics, or muscle relaxants. It is recommended that drugs with the shortest half-lives be used [6]. Regional techniques are encouraged when applicable. Bilevel positive airway pressure (BIPAP) or continuous positive airway pressure (CPAP), when tolerated by the patient, provides supplementary ventilatory support in patients without an existing tracheostomy.

Central nervous system (CNS) stimulants such as caffeine and doxapram are known to trigger the respiratory center, increasing respiratory rate, and improving ventilation. Caffeine was studied with the purpose of reducing apnea in premature infants [7,8]. Caffeine is a methylxanthine derivative that acts by inhibiting adenosine receptors with concomitant nonselective phosphodiesterase inhibition [9,10]. Observed caffeine effects include increased catecholamine release; promotion of wakefulness via central stimulation; enhancement of diaphragmatic contractility via adrenal release of epinephrine; augmentation of peripheral chemoreceptor activity and sustained diaphragmatic activity with associated tidal volume increases in preterm infants [10]. There is also a suggested role for caffeine in the antagonism of the depressant effects of opioids [9].

Currently, there are no medications that completely reverse the unconscious states induced by general anesthesia. However, caffeine usage in both rat and human models has demonstrated an acceleration of emergence from isoflurane and propofol anesthesia [11-13]. Wang et al. found that when compared to theophylline and forskolin, caffeine had the highest efficacy at accelerating emergence from anesthesia [13]. In humans, caffeine leads to a more rapid emergence due to the elevation of ionized cyclic adenosine monophosphate and minimally due to the blockade of the adenosine receptor [12-14]. Fox et al. found that administration of caffeine resulted in a mean time to emergence of 9.6 minutes, while placebo demonstrated a mean time to emergence of 16.5 minutes, which was statistically significant with a p-value of 0.002 [13]. This corresponded to a 42% reduction in time to emergence when caffeine was administered 10 minutes before case end.

Caffeine usage is well tolerated but may have various dose-related side effects. These side effects include altered sleep-wake cycles, tremors, agitation, irritability, tachycardia, tachypnea, hypertonia, and tonic-clonic movements [7,9]. According to the caffeine for apnea of prematurity trial, caffeine is associated with decreased weight gain in the pediatric population but no significant short-term or long-term consequences [14]. Subsequent randomized controlled trials using high-dose caffeine therapy demonstrated no major sequelae to caffeine use [9]. Benefits of caffeine includes cost reductions, reduced mechanical ventilator days, clinically significant reduction in episodes of postoperative apnea and bradycardia, and neuroprotection in premature infants [7,9]. Overall, caffeine offers accelerated recovery from general anesthesia and a decreased incidence of post-extubation respiratory events including desaturation events, breath holding, laryngospasms, obstruction, and reintubation [10].

In this case, caffeine was administered and titrated to achieve spontaneous ventilation with an EtCO_2_ of 62 mmHg, close to the patient’s baseline ETCO_2_. Overcorrecting this patient’s chronic hypercapnia could have led to alkalemia, a paradoxical reduction in respiratory drive, and may have induced neurological dysfunction including seizures. Given the concerns of overcorrecting his hypercarbia, we opted to bolus increments of caffeine instead of a higher loading dose. This method allowed for observing the response of caffeine to the respiratory mechanics in a caffeine-naive patient. The patient became alert with purposeful movements and met tracheal extubation criteria after these efforts were made.

## Conclusions

Given a history of prolonged intubation and unplanned hospital stays, this patient had a planned PICU stay postoperatively; however, this was his shortest overall hospital stay after being administered an anesthetic. Overall, the only major deviation from previous efforts was the addition of intravenous caffeine. In patients with CCHS, caffeine usage can allow for a shortened postoperative course by improving alertness and ventilation, allowing for tracheal extubation immediately following lengthy procedures, and minimizing postoperative respiratory events.
